# Effectiveness of Telerehabilitation in Persons With Spinal Cord Injury During the COVID-19 Pandemic (TELE-SCOPE): A Single-Center, Double-Blind, Randomized Controlled Trial

**DOI:** 10.7759/cureus.41513

**Published:** 2023-07-07

**Authors:** Raktim Swarnakar, Shivlal Yadav, Sanjay Wadhwa, Srikumar Venkataraman

**Affiliations:** 1 Physical Medicine and Rehabilitation, All India Institute of Medical Sciences, New Delhi, New Delhi, IND

**Keywords:** coronavirus pandemic, physical medicine and rehabilitation, spinal cord injury, telerehabilitation, covid-19

## Abstract

Introduction

The COVID-19 pandemic has posed numerous challenges in accessing adequate healthcare services, particularly for individuals with spinal cord injury (SCI). On the other hand, telerehabilitation has emerged as a promising solution to address healthcare needs. Since there was no study during the pandemic, we started this study with the aim of assessing the efficacy of telerehabilitation for individuals with SCI during the COVID-19 pandemic.

Methods

This is a prospective double-blind, randomized, controlled trial conducted in a tertiary rehabilitation care center hospital. Thirty participants with traumatic spinal cord injuries (age 18 years or more, either gender) were equally randomized to the telerehabilitation or control group (1:1). Biweekly telerehabilitation sessions (each session: 30 minutes) were provided. Participants in the control group were advised to continue standard usual care as advised previously during outpatient or inpatient rehabilitation. The Spinal Cord Independence Measure (SCIM III) (primary outcome measure) and Coronavirus Anxiety Scale (CAS) (secondary outcome measure) were evaluated at baseline, four weeks, and eight weeks.

Results

The mean age of the intervention group was 28.2±6.9 years, and the mean age of the control group was 26.3±7.7 years. The self-care (P = 0.03) and mobility domains (P=0.01) of the SCIM III in the intervention group compared to the control group, as determined through a between-group analysis, showed statistically significant differences. CAS also showed improvement in the intervention group compared to the control group. Within-group analysis showed a mean difference of 6.3 points in the intervention group compared to the control group (1.3 points).

Conclusion

Telerehabilitation intervention is safe, feasible, and effective in improving self-care and mobility domains in persons with spinal cord injuries during the pandemic. It is also effective in reducing the anxiety related to the coronavirus in this population. Further research with a larger sample size and a longer duration is needed to evaluate long-term effectiveness during such crises.

## Introduction

Rehabilitation is not just a mere component of healthcare; it is a fundamental need for individuals with spinal cord injuries (SCI). SCI can result in severe physical limitations, affecting mobility, sensation, and bodily functions. Therefore, effective rehabilitation is essential to optimize recovery and enhance the overall well-being of these individuals.

However, the onset of the global pandemic presented unprecedented challenges, disrupting traditional healthcare systems and posing immense barriers to accessing in-person rehabilitative services. Lockdowns, travel restrictions, and social distancing measures imposed to curb the spread of the virus significantly impacted the ability of individuals with SCI to receive the care they desperately needed.

In response to this predicament, telemedicine emerged as a promising alternative for delivering healthcare services remotely. Telerehabilitation, a specific branch of telemedicine, enables healthcare providers to connect with their patients virtually, ensuring continuity of care and facilitating rehabilitation interventions [1-3]. Through telerehabilitation, individuals with SCI could engage in remote consultations, receive guidance on exercises and therapy, and even access educational resources tailored to their specific needs [4]. The benefits of telerehabilitation for individuals with SCI became even more evident during the height of the pandemic, when healthcare facilities were overwhelmed and in-person visits were restricted or discouraged. Telerehabilitation not only ensured the safety of patients and healthcare providers by reducing the risk of viral transmission but also offered convenience and flexibility, eliminating the need for extensive travel and allowing individuals to receive care from the comfort of their homes. However, as the pandemic persisted and new variants of the virus emerged, it became apparent that telerehabilitation was not merely a temporary solution but a critical component of the future of healthcare delivery. The waves of the pandemic continue to pose uncertainties, making it imperative to evaluate and understand the effectiveness of telerehabilitation in the current times. Furthermore, telerehabilitation is known to be effective in improving functional status in many chronic diseases, but it has often shown insufficient evidence [3,4]

Therefore, this proposed study aims to delve deeper into the efficacy of telerehabilitation in addressing the rehabilitation needs of individuals with SCI. By examining the outcomes, patient experiences, and overall satisfaction with telerehabilitation, we can provide valuable insights into the feasibility and benefits of this approach. This study will contribute to the growing body of evidence supporting telemedicine as an integral part of universal health coverage, particularly for those with specific healthcare requirements such as SCI.

## Materials and methods

Ethics

The study protocol (conformed to Standard Protocol Items: Recommendations for Interventional Trials (SPIRIT)) was approved by the Institutional Review Board (IRB) of the All India Institute of Medical Sciences (AIIMS), New Delhi, India (IECPG-676/25.08.2022, RT-45/29.09.2022). All study procedures were done in accordance with the 2013 revised Helsinki Declaration and the Good Clinical Practice guidelines of the International Conference on Harmonization. All participants were provided a written informed consent form before enrollment in the study. This study was also prospectively registered on the Clinical Trial Registry-India (CTRI) with the unique identification number CTRI/2022/10/046657.

Study design and setting

This was a prospective, double-blinded, randomized controlled trial (TELE-SCOPE trial) conducted from the 28th of October 2022 to the 31st of May 2023 in the rehabilitation care setting.

Study participants

All cases of traumatic spinal cord injury (SCI) previously diagnosed and followed up on or who got outpatient or inpatient rehabilitation were recruited. Inclusion criteria were: age 18 years or older; either gender; traumatic or non-traumatic SCI who received inpatient or outpatient rehabilitation before or during the outbreak of COVID-19. Exclusion criteria were: no access to or unavailability of the minimum phone-calling facility; any kind of emergency condition requiring hospitalization; progressive deterioration of a neurological condition; and not being willing to participate in the study.

Randomization and blinding

Participants were randomized to both groups (1:1) via a remote computer-based randomization system, which ensured the concealment of the allocation sequence. The randomization sequence list was made in advance of recruitment by a research assistant who was not a part of the research team. Double-blinding was done according to previously published literature [5]. The evaluator and patients were blinded during the entire study. The evaluator was blinded to the study objectives, randomization of patients’ groups, and sequence of randomization. Patients were blinded to which group they were allocated and were unaware of other treatment modalities.

Consent

A participant informed consent form (PICF) (in English and Hindi) was sent electronically to the participants. After reading the form, participants were asked whether they agreed or disagreed to participate in the study; if they agreed, they were asked to send a text message stating, "Yes, I consent to take part in the concerned study" at the beginning of the telephone interview. Participants could also send an email, audio, or video message. The informed consent thus given by the participants was documented or recorded. This procedure of taking informed consent was in accordance with the Telemedicine Practice Guidelines, India (March 25, 2020) [6].

Interventions

Telerehabilitation group (group A): biweekly telerehabilitation sessions were provided. Each session was of 30 minutes duration [4,7]. At the end of four and eight weeks, evaluations with outcome measures were performed. Each session included the following things: physiotherapy (PT) and occupational therapy (OT) demonstrations; addressing current concerns; listing out routine problems faced during activities of daily living; addressing clinical complaints; and following advice according to the evidence-based recommendations for SCI people. COVID-19-specific recommendations and precautions for SCI people were given. Hygienic practices during a pandemic were also advised according to recent guidelines [8,9]. Physiotherapy techniques like range of motion, stretching, and strengthening exercises (demonstrated via a telerehabilitation facility) were followed according to the particular patient's needs. Participants were advised to follow those exercises two times a day (30 minutes each time). OT was also followed, and participants were advised to continue it two times a day (30 minutes each time).

Control group (group B): They were advised to continue standard usual care (as patients usually receive PT and OT during in-person visits to hospitals) as advised previously during outpatient or inpatient rehabilitation. At baseline and at the end of four and eight weeks, an evaluation with outcome measures was performed.

Data monitoring and safety

A data and safety monitoring board was formed and could decide to withdraw or remove a study participant if there were any serious adverse events as a result of the trial. The board members were accessible by telephone to all participants throughout the study.

Study outcome measures

The primary outcome measure was improvement in the Spinal Cord Independence Measure (SCIM III), and the secondary outcome measure was improvement in the Coronavirus Anxiety Scale (CAS) [10,11]. SCIM III and CAS were evaluated at baseline, four weeks, and eight weeks before the concerned 30-minute session that day.

Sample size

During the COVID-19 pandemic, there was no randomized controlled trial (RCT) in spinal cord injury patients with telerehabilitation. Moreover, previously, there was no study with such an intervention and outcome measure in SCI. In completely different circumstances with selected outcome measures, how feasible and effective the intervention would be is not known. As previous research showed, the sample size for any pilot RCT should be 20, 24, 30, or 40 [12]. We anticipate that our pilot RCT with telerehabilitation in SCI during the COVID-19 pandemic would help in finding an accurate sample size for future definitive studies. As it was planned for a pilot RCT, we aimed for 30 participants.

Statistical analysis

Data were entered in a Microsoft Excel spreadsheet (Microsoft Corporation, New York, USA), and statistical analysis was done using Stata 14 (StataCorp LLC, College Station, USA). Data were presented as mean±SD/median (range, min, max), and frequency percentage. An intention-to-treat (ITT) analysis was carried out. Categorical variables were compared by Chi-squared or Fisher's exact test. Continuous variables were compared by independent t-test, and the difference between the two groups was reported with a 95% confidence interval. On the other hand, the Wilcoxon rank sum test compared the continuous variables that did not follow the normal distribution. A P value <0.05 was considered statistically significant.

## Results

Study participants

**Figure 1:**
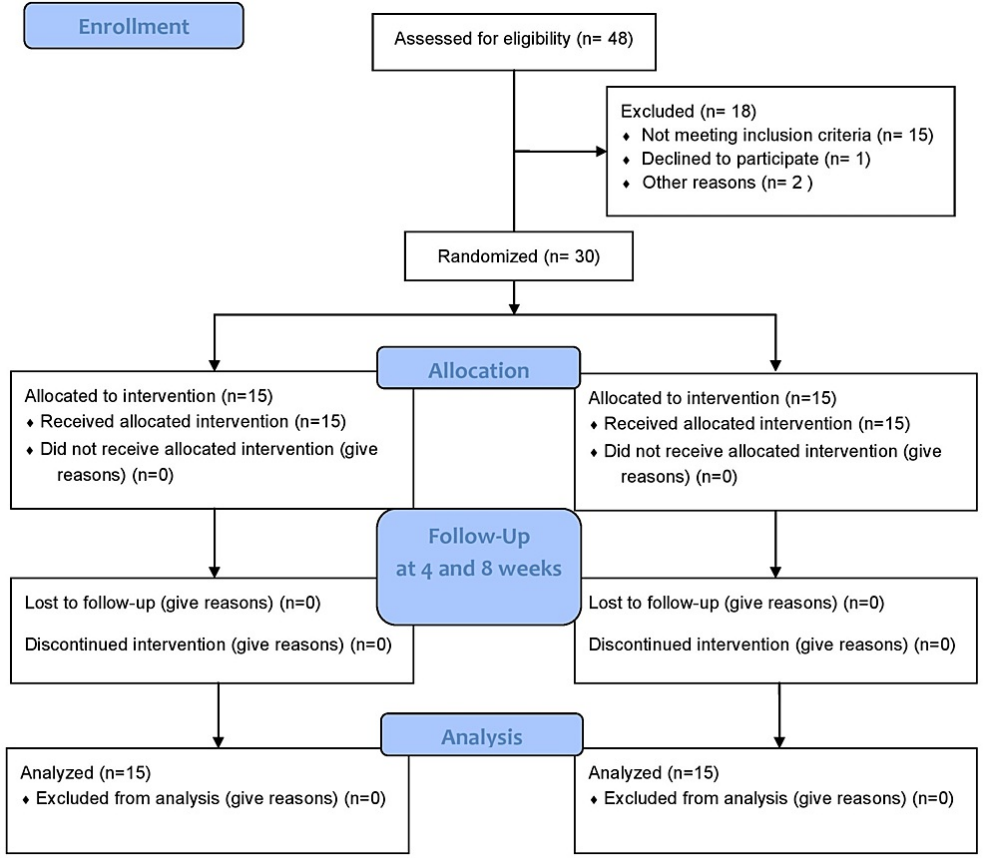
CONSORT flow diagram. CONSORT: consolidated standards of reporting trials; n: number.

Figure 1 shows the study flow diagram. We screened 48 eligible participants, and 18 were excluded. The 30 participants who met the eligibility criteria were randomly assigned: 15 participants were assigned to receive telerehabilitation, and 11 participants were assigned to the control group. All study participants completed the eight weeks of treatment. 

Baseline characteristics of the patients

Among 30 participants (mean±SD age: 27.3±7.2 years), the majority were males (Table 1). The majority were paraplegics with complete injuries, and only one was tetraplegic. Details of other baseline characteristics are mentioned in Table 1.

**Table 1:** Baseline demographic and clinical characteristics. SD: standard deviation; n (%): number (percentage); IQR: interquartile range; NLI: neurological level of injury; AIS: ASIA (the American Spinal Injury Association) Impairment Scale.

Characteristics	Group A (telerehabilitation group) (N=15)	Group B (control group) (N=15)	Total (N=30)	P-value
Age (years), mean±SD	28.2±6.9	26.3±7.7	27.3±7.2	0.49
Sex, n (%)				0.59
Male	12 (80%)	14 (93.3%)	26 (86.7%)	
Female	3 (12%)	1 (6.7%)	4 (13.3%)	
Education, n (%)				1.00
Graduated	8 (53.3%)	8 (53.3%)	16 (53.3%)	
Not graduated	7 (46.7%)	7 (46.7%)	14 (46.6%)	
Duration of Injury (months), median (IQR)	18.66 (9-24)	17.66 (5-18)	18.16 (6-18)	0.21
NLI, n (%)				Not applicable
Cervical	0 (0%)	1 (6.7%)	1 (3.3%)	
High thoracic	6 (40%)	2 (13.3%)	8 (26.7%)	
Low thoracic	7 (46.7%)	9 (60%)	16 (53.3%)	
Lumbar	2 (13.3%)	3 (20%)	5 (16.7%)	
AIS, n (%)				0.33
Complete=A	14 (93.3%)	11 (73.3%)	25 (83.3%)	
Incomplete=B	1 (6.7%)	3 (20%)	4 (13.3%)	
Incomplete=D	0 (0%)	1 (6.7%)	1 (3.3%)	

Primary outcome

Table 2 shows the comparison of all domains of SCIM III. 

**Table 2:** Comparison of SCIM III between two groups. SD: standard deviation; SCIM: Spinal Cord Independence Measure; CI: confidence interval.

Variables	Group A (telerehabilitation group) (N=15), mean±SD	Group B (control group) (N=15), mean±SD	Difference (95% CI)	P-value
SCIM III: self-care domain				
Baseline	3.6±1.4	3.7±1.4	−0.06 (−1.13 to 0.99)	0.89
Four weeks	4.0±1.4	3.9±1.2	0.13 (−0.82 to 1.09)	0.77
Eight weeks	5.3±1.9	4.0±1.1	1.27 (0.11 to 2.42)	0.03
SCIM III: respiration and sphincter management domain				
Baseline	22.4±4.9	22.6±3.9	−0.2 (−3.53 to 3.13)	0.90
Four weeks	23.5±4.6	22.9±3.8	0.53 (−2.61 to 3.68)	0.73
Eight weeks	24.5±4.9	23.3±3.8	1.27 (2.03–4.57)	0.44
SCIM III: mobility domain				
Baseline	3.6±1.7	3.8±1.6	−0.2 (−1.43 to 1.03)	0.74
Four weeks	4.9±1.8	4.0±1.4	0.86 (−0.36 to 2.09	0.16
Eight weeks	6.1±1.9	4.3±1.4	1.73 (0.46–2.99)	0.01
SCIM III: total				
Baseline	29.5±6.2	30.2±5.2	−0.7 (−5 to 3.5)	0.730
Four weeks	32.4±5.8	30.8±5.0	1.5 (−2.5 to 5.6)	0.447
Eight weeks	35.8±7.1	31.6±4.9	4.2 (−0.34 to 8.8)	0.068
Difference (95% CI) within-group baseline and eight weeks	−6.3 (−9.0 to −3.6) P-value=0.002	−1.3 (−2.2 to −0.4) P-value=0.006		

Secondary outcome

Table 3 shows the between-group comparison of CAS. 

**Table 3:** Comparison of CAS between two groups. SD: standard deviation; CAS: Coronavirus Anxiety Scale; CI: confidence interval.

Variables	Group A (telerehabilitation group) (N=15), mean±SD	Group B (control group) (N=15), mean±SD	Difference (95% CI)	P-value
CAS				
Baseline	10.4±1.4	10.3±2.1	0.06 (1.26–1.39)	0.91
Four weeks	10.1±1.4	10.3±2.1	0.2 (1.54–1.14)	0.76
Eight weeks	9.5±1.4	9.9±1.6	0.4 (1.52–0.72)	0.47

Safety, feasibility, and adherence

About 100% of the randomized participants completed the eight weeks of the trial duration. There were no dropouts or 100% adherence to the prescribed rehabilitation program. No participants reported any adverse events related to any interventions in either group.

New complications

In the intervention group, we observed new-onset pressure injuries (PI) in four out of 15 participants (26.6%). However, in the control group, there were multiple complications that arose, including new-onset pressure injuries, increased spasticity, muscle spasms, and a higher incidence of urinary tract infections. Specifically, in the control group, 11 out of 15 participants (73.3%) experienced these complications. Analysis showed the difference in incidences of PI in both groups was not statistically significant (total PI incidences in the telerehabilitation group: 26%, in the control group: 53%, P=0.26). But when compared in terms of total complications, it was found to be statistically significant (total complications in the telerehabilitation group: 26%, in the control group: 73%, P=0.02).

Economic assessment

Travel costs were completely eliminated in group A. Total travel expenses calculated as: travel cost for monthly visits to the hospital for two months of study duration (35.64±37.98 (mean±SD, unit: USD)). The cost of using the internet (6.86±0.49 USD) was much less than the cost of traveling (5-10 times, P=0.01). The internet cost was calculated as the monthly internet subscription price multiplied by two months.

## Discussion

This is the first study to evaluate the effectiveness of telerehabilitation for SCI during the pandemic. Results of our study showed that eight weeks of telerehabilitation intervention during the pandemic were safe, feasible, and cost-effective for people with SCI.

An approach increasingly being used to extend access to care in low- and middle-income countries, particularly when care delivery is challenged and in remote geographical locations [1,4], is telemedicine. Telemedicine involves using information and communication technologies to provide care and education [2]. Telerehabilitation is a subset of telemedicine, defined as the provision of rehabilitation services at a distance using telecommunication technology [3].

The pandemic has caused disruptions in health services around the globe. People with SCI face more issues than able-bodied individuals. Telemedicine started to prosper during this pandemic to cater to health services effectively [4]. There was no trial of telerehabilitation for SCI during the pandemic. Previous trials looked only at the status of pressure injuries and quality of life [13,14].

There were one RCT and one case series from India [13,14], and one from Bangladesh [15]. These studies were conducted during pre-covid times in people with SCI. Some showed improvement following telerehabilitation [13], and some showed no statistically significant difference [15]. But intervention types and durations were different from those in our study. Furthermore, no RCT from India used the Spinal Cord Independence Measure (SCIM III) as their outcome measure, which gives a more detailed evaluation. 

In our study, we observed a significant improvement in the self-care and mobility domains of the Spinal Cord Independence Measure III (SCIM III) in the intervention group (after eight weeks of intervention) compared to the control group, as determined through a between-group analysis (Table 2). This means that the participants who received the intervention showed a notable enhancement in their ability to take care of themselves and move independently, in contrast to those in the control group.

Additionally, we found statistically significant differences (intervention group: P=0.002, control group: P=0.006) in both groups when we conducted a within-group analysis (Table 2). This suggests that there were improvements within each group separately. However, it is important to note that the mean difference in the intervention group was 6.3, while in the control group it was 1.3. This indicates a substantial clinical difference between the two groups, with the intervention group experiencing a much greater improvement in SCIM III total compared to the control group (Table 2).

Furthermore, the Coronavirus Anxiety Scale (CAS) was developed to assist clinicians and researchers in efficiently identifying individuals who are functionally impaired due to coronavirus-related anxiety. When we conducted an analysis comparing the intervention group to the control group, the CAS showed a significant difference of 0.4 points at the eight-week mark. According to the CAS (Table 3), the intervention group experienced a greater reduction in coronavirus-related anxiety than the control group.

In our study, we found compelling evidence of a significant cost reduction in the intervention group (P=0.01), specifically related to travel expenses. This means that individuals who received the intervention incurred significantly lower transportation costs compared to those in the control group. The intervention likely provided some form of support or alternative means to minimize travel requirements, resulting in financial savings for the participants.

Moreover, our study also revealed a noteworthy reduction in new-onset complications within the intervention group. This implies that individuals who underwent the intervention experienced a lower incidence of newly developed complications or health issues compared to the control group. The specific nature of the intervention may have contributed to preventing or minimizing the occurrence of these complications, thus demonstrating its effectiveness in promoting better health outcomes. The findings from our study also corroborate the existing literature [16,17]. Recent systematic reviews and meta-analyses also revealed that, though telerehabilitation is found to improve certain domains of neurological disorders, it needs more evidence [18]. Particularly in people with SCI, evidence with telerehabilitation is similarly lacking [19,20].

Limitation

Our study had a small sample size as it was conducted as a pilot intervention. This limited sample size may have hindered our ability to detect statistically significant differences in certain domains of the SCIM III. Additionally, due to the relatively short duration of the intervention, which lasted eight weeks, we were unable to assess the long-term effectiveness of telerehabilitation interventions during the pandemic. Therefore, it is important to interpret the results of our study with caution, considering these limitations.

Future research

Further research with larger sample sizes and longer intervention durations is warranted to provide a more comprehensive understanding of the effects and long-term outcomes of telerehabilitation interventions in the context of a pandemic or any natural disasters or crisis.

## Conclusions

In conclusion, this study is the first to evaluate the effectiveness of telerehabilitation in individuals with spinal cord injury (SCI) during the pandemic. The results indicate that telerehabilitation interventions during the pandemic were safe, feasible, and cost-effective for people with SCI.

The study demonstrated significant improvements in self-care and mobility among participants who received the telerehabilitation intervention for eight weeks compared to the control group. There were also reductions in coronavirus-related anxiety and travel expenses in the intervention group. Additionally, a decrease in new-onset complications was observed in the intervention group. However, future research should include larger sample sizes and longer intervention durations to further explore the effects and long-term outcomes of telerehabilitation interventions during the pandemic. Overall, this study provides important evidence supporting the effectiveness of telerehabilitation for individuals with SCI during the pandemic. It highlights the potential of telemedicine and telerehabilitation to address healthcare challenges and improve functional outcomes, particularly in times of crisis.
